# Perilipin 4 in human skeletal muscle: localization and effect of physical activity

**DOI:** 10.14814/phy2.12481

**Published:** 2015-08-11

**Authors:** Shirin Pourteymour, Sindre Lee, Torgrim M Langleite, Kristin Eckardt, Marit Hjorth, Christian Bindesbøll, Knut T Dalen, Kåre I Birkeland, Christian A Drevon, Torgeir Holen, Frode Norheim

**Affiliations:** 1 Department of Nutrition, Institute of Basic Medical Science, Faculty of Medicine, University of OsloOslo, Norway; 2 Department of Endocrinology, Morbid Obesity and Preventive Medicine, Oslo University Hospital and Faculty of Medicine, University of OsloOslo, Norway

**Keywords:** Endurance training, lipid droplets, phosphatidylcholine, phosphatidylethanolamine, PLIN4, sarcolemma, skeletal muscle fibre

## Abstract

Perilipins (PLINs) coat the surface of lipid droplets and are important for the regulation of lipid turnover. Knowledge about the physiological role of the individual PLINs in skeletal muscle is limited although lipid metabolism is very important for muscle contraction. To determine the effect of long-term exercise on PLINs expression, 26 middle-aged, sedentary men underwent 12 weeks combined endurance and strength training intervention. Muscle biopsies from *m. vastus lateralis* and subcutaneous adipose tissue were taken before and after the intervention and total gene expression was measured with deep mRNA sequencing. *PLIN4* mRNA exhibited the highest expression of all five PLINs in both tissues, and the expression was significantly reduced after long-term exercise in skeletal muscle. Moreover, *PLIN4* mRNA expression levels in muscle correlated with the expression of genes involved in *de novo* phospholipid biosynthesis, with muscular content of phosphatidylethanolamine and phosphatidylcholine, and with the content of subsarcolemmal lipid droplets. The PLIN4 protein was mainly located at the periphery of skeletal muscle fibers, with higher levels in slow-twitch as compared to fast-twitch skeletal muscle fibers. In summary, we report reduced expression of PLIN4 after long-term physical activity, and preferential slow-twitch skeletal muscle fibers and plasma membrane-associated PLIN4 location.

## Introduction

Obesity is caused by a positive imbalance between energy intake and expenditure promoting adipose tissue hypertrophy and increased ectopic accumulation of lipid droplets (LDs) in other tissues like skeletal muscle (De Ferranti and Mozaffarian 2008; Webber 2008; Glatz et al. 2010). Several reports show a positive correlation between intramyocellular (IMC) accumulation of LDs and insulin resistance in sedentary obese and type 2 diabetic subjects (Pan et al. 1997; Goodpaster et al. 2000; Levin et al. 2001). However, the association between insulin sensitivity and amounts of LDs (Thamer et al. 2003) is confounded by the fact that both endurance-trained athletes and obese subjects may have increased IMC triacylglycerol levels (Tauchi-Sato et al. 2002) despite being insulin sensitive or insulin resistant, respectively (Goodpaster et al. 2001; van Loon et al. 2003).

LDs are cellular organelles with a core of neutral lipids sequestered by a monolayer of phospholipids imbedded with LD-binding proteins (Cermelli et al. 2006; Greenberg et al. 2011). LDs in skeletal muscle fibers are distributed from the subsarcolemmal region (SS) to the centre of the intermyofibrillar (IMF) region (Palmer and Hoppel 1986). Recently, we demonstrated that the volume of LDs in the SS region was reduced in men completing 12 weeks of combined strength- and endurance training, whereas LD size in the IMF region was unchanged by long-term physical activity (Li et al. 2014).

The size and number of intracellular LDs depend on lipid availability and activity of enzymes and co-activators required for synthesis and degradation of LDs. Many of these enzymes have to be present at the LD surface to be enzymatically active. Proteins on the LD surface vary among cell types but members of the perilipin (PLIN) family have been identified as particularly abundant LD-binding proteins across mammalian cell types and species (Zhang et al. 2011; Na et al. 2013). So far, five genes (*PLIN1-5*), some of which encode more than one protein, have been identified based on family-specific sequence homology (Kimmel et al. 2010). These PLIN proteins are tissue specifically expressed (Brasaemle 2007). PLIN1 is mainly expressed in adipocytes and steroidogenic cells (Greenberg et al. 1991; DA Servetnick et al. 1995), while PLIN2 and 3 are expressed in most tissues (Brasaemle et al. 1997; Dalen et al. 2004). Expression of PLIN4 seems to be limited to adipose cells, brain, skeletal muscle, and heart (Wolins et al. 2003; Dalen et al. 2004), and PLIN5 is expressed in oxidative tissues like skeletal muscle (Wolins et al. 2006; Yamaguchi et al. 2006; Dalen et al. 2007). Except for *PLIN3*, expressions of the *PLIN* genes are affected by changes in the concentration of circulating fatty acids (FAs) by their ability to activate peroxisome proliferator-activated receptors (PPARs) (Dalen et al. 2004, 2006; Bindesboll et al. 2013).

PLIN1 was the first PLIN to be identified and cloned (Greenberg et al. 1991, 1993) and is the best characterized member of the family. Its presence on adipose LDs regulates recruitment of lipases along with co-activators to the LD surface. PLIN1 is fundamental in the regulation of storage versus degradation of neutral lipids stored in the LD core (Martinez-Botas1 et al. 2000; Tansey et al. 2001; Sztalryd et al. 2003; Bickel et al. 2009; Beylot et al. 2012). When cultured adipocytes are incubated with lipids, the coating of nascent LDs with PLIN4 permits packaging of newly synthesized triacylglycerol (TAG) (Wolins et al. 2003). This suggests that PLIN4 participate early in the formation of LDs in adipocytes. PLIN2-5 is believed to compartmentalize and protect neutral lipids in the LD core from degradation in nonadipose tissues. Some PLINs exhibit strong association to intracellular LDs based on the types of stored neutral lipids (Hsieh et al. 2012). For example, whereas PLIN5 exhibits strong preference for localization to LDs containing TAG, PLIN4 prefers LDs containing cholesterol esters (CE) (Hsieh et al. 2012). This shows that a single cell can contain pools of physically and biochemically distinct LDs, where the type of PLIN(s) expressed favor selective accumulation of certain neutral lipids. Several PLINs may change expression in response to physical activity (Gjelstad et al. 2012). Although the level of PLIN5 protein is increased by endurance training in several studies (Peters et al. 2012; Louche et al. 2013; Shepherd et al. 2013), the effect of training on PLIN2 and 3 expression is more inconsistent (Peters et al. 2012; Shaw et al. 2012; Louche et al. 2013; Shepherd et al. 2013; Covington et al. 2014). Two studies reported no significant effect of endurance training on PLIN4 expression in humans (Gjelstad et al. 2012; Peters et al. 2012).

Due to the inconsistent findings regarding PLIN expression in response to exercise, we studied PLIN expression in men undergoing a 12 weeks carefully controlled intervention with combined strength- and endurance training. We observed a reduction in *PLIN4* mRNA in *m*. *vastus lateralis* after completion of the training period. Furthermore, using cell fractionation and immune staining, we provide evidence suggesting that the majority of PLIN4 is located in the SS-region. These findings link our previously reported reduction in LDs in the SS-region after training (Li et al. 2014) to the expression level of the LD-binding protein *PLIN4*.

## Material and Methods

### Ethics approval

The study adhered to the Declaration of Helsinki and was approved by the National Regional Committee for Medical and Health Research Ethics North, Tromsø, Oslo, Norway. The study was registered with the US National Library of Medicine Clinical Trials registry (NCT01803568). Written informed consent was obtained from all participants prior to any study-related procedure.

### Exercise intervention

A training intervention study was performed as described elsewhere (Norheim et al. 2014). Healthy sedentary men (*n* = 26; 40–65 years of age) were recruited. The participants had an average body mass index (BMI) of 26.5 kg/m^*2*^ (min 20.9, max 32.5), and 11 subjects had fasting glucose concentration ≥5.6 mmol/L and/or impaired OGTT (2 h serum glucose concentration ≥7.8 mmol/L). The participants were subjected to a combined strength- and endurance-training program for 12 weeks, including two endurance bicycle sessions (60 min) and two whole-body strength-training sessions (60 min) per week. Each endurance session started with a 10 min warm-up at three different workloads, corresponding to 50% (4 min), 55% (3 min), and 60% (3 min) of maximum oxygen uptake (VO_2_max).

### Human biopsies

Biopsies from *m*.* vastus lateralis* were taken before as well as after 12 weeks of training using Bergstrom needles (Bergstrom 1962). Muscle biopsies from 26 subjects were quickly rinsed in ice-cold PBS and dissected on PBS-soaked filter paper on a cold aluminum plate under a stereo magnifier to remove visible fat, blood and connective tissue. Each sample was divided into small pieces for EM, immunohistochemistry and RNA isolation. Tissue for RNA isolation was immediately transferred to RNA-later (Qiagen, Limburg, The Netherlands) overnight, which was drained off before storing the tissue at −80°C. EM-selected muscle bundles were submerged in cold fixative (2% formaldehyde (w/v), 2% glutaraldehyde (v/v) in 0.1 mol/L sodium phosphate buffer, pH 7.4) and stored at 4°C for 2–6.5 h before osmification; muscle bundles were soaked in 1% OsO4 (w/v) in 0.1 M sodium phosphate buffer for 30–35 min with continual rotary motion. Osmificated samples were embedded in Durcupan (Fluka, Sigma-Aldrich Chemie GmbH, Steinheim, Switzerland). For immunohistochemistry, muscle fibers were fixed in 4% paraformaldehyde (w/v), 0.025% glutaraldehyde (v/v) in PBS for 4–6 h at room temperature. Then, samples were stored in a 1:10 dilution of fixative at 4°C until paraffin embedding.

A subcutaneous adipose tissue biopsy was taken from the periumbilical region ∼30 min after the bicycle session, before as well as after 12 weeks of training. Biopsies were frozen immediately in liquid nitrogen and stored at −80°C until further processing.

### Preparation of skeletal muscle biopsy for lipidomics

Skeletal muscle biopsies were accurately weighed; freeze-dried and stored at −80°C. At the day of analysis the samples were thawed and homogenized in chloroform, methanol and water using a motorized pellet pestle (Kontes; Vineland, NJ). After centrifugation the supernatant was analyzed for lipid class content using an Agilent 1100 normal phase liquid chromatography system coupled to an Evaporative Light Scattering Detector (ELSD). Separation of the neutral lipids was performed on a Chromolith Performance Si 100–4.6 mm high-performance liquid chromatography (HPLC) column from Merck using a mixture of hexane, MTBE, and acetic acid as mobile phase. The polar lipids were separated on an YMC PVA-SIL-NP 250x 4.6 mm, 5 *μ*m column from YMC using hexane, isopropanol, acetonitrile, chloroform, water, MTBE, and acetic acid as mobile phase. Unknowns were calibrated against known standards from NU-CHEK-PREP, INC, and reported as g /100 g sample.

### Animal experiments

Use of animals was approved and registered by the Norwegian Animal Research authority. Mice were housed in a temperature-controlled (22°C) facility with a strict 12 h light/dark cycle. Male C57BL/6N mice (Charles River) were fed ad libitum a standard chow diet and euthanized by cervical dislocation at the onset of the light cycle (17 weeks). Gastrocnemius and soleus muscles were dissected and snap frozen in liquid nitrogen. The tissue was stored at −80°C until gene expression analysis was conducted. RNA was isolated as previously described (Bindesboll et al. 2013).

### Analysis of LDs

Details concerning EM imaging and LDs quantification have been described in details elsewhere (Li et al. 2014). Briefly, EM images from Durcupan blocks (sampled before and after intervention) were taken at 4200-fold magnification using a Tecnai G2 electron microscope from FEI (Hillsboro, OR). Twenty EM images from both the SS and IMF region were randomly chosen for software-assisted analysis (Science Linker, Science Linker As, Oslo, Norway) (Figure S1). Each LD was manually identified and marked. From images of the SS and IMF regions the boundaries of the two regions were marked manually and the software calculated the area sizes. The area of each LD was calculated from the measured LD diameter as *π*r^2^. The total area of LDs was calculated as the sum of all LD areas and presented relative to SS and IMF region area, respectively. Measurement of SS area and fiber width by direct tracing methods provided data for quantification of the SS space fraction. The SS area percentage was calculated as the ratio of the SS areas divided by total muscle section area at each cross section.

### Immunohistochemical analyses

Paraffin-embedded muscle fibers were cut into 5 *μ*m sections using a rotary microtome (HM 355 S, MICROM International GmbH, Walldorf, Germany) and mounted on Superfrost Plus glass slides (Thermo Scientific, Waltham, MA). The sections were incubated over night at 37°C, and deparaffinized in xylene (Sigma-Aldrich) for 2 × 5 min. Sections were rehydrated stepwise in 100%, 96%, 70%, and 50% ethanol for 5 min each before a final rinse in tap water for 5 min. The slides were subjected to heat-induced antigen retrieval by heating in 1 mmol/L EDTA (pH 8) at 95°C for 15 min, and cooling to room temperature for 20 min. Next, the muscle sections were incubated in blocking solution 1 (0.01 mol/L PBS containing 0.05% Triton X-100 (v/v), 1% BSA (w/v), and 3% newborn calf serum (NCS) (v/v) at room temperature for 1 h followed by overnight incubation at 4°C with primary antibody diluted in blocking solution 2 (0.01 mol/L PBS containing 0.05% Triton X-100, 1% BSA, and 10% NCS). The following primary antibodies were used: rabbit anti-PLIN4 1:100 (#HPA044682, Sigma Life Science, MO), guinea pig anti-PLIN4 1:100 (#GP34, Progen, Heidelberg, Germany), rabbit anti-dystrophin 1:100 (#ab15277, Abcam, Cambridge, UK), mouse anti-myosin heavy chain (skeletal, fast) 1:1000 (#M1570, Sigma), guinea pig anti-PLIN5 1:100 (#GP31, Progen), and mouse anti-PLIN1 1:2 (#651156, Progen). Then, sections were incubated with fluorochrome-conjugated secondary antibodies diluted 1:400 in blocking solution 2. Finally, muscle sections were incubated with 0.5 *μ*g/mL Hoechst 33342 (Sigma-Aldrich) diluted in PBS. The muscle sections were rinsed with PBS between each incubation step. The slides were mounted with coverslips (Gerhard Menzel Glasbearbeitungswerk, Braunschweig, Germany) using Dako mounting medium (DakoCytomation, Glostrup, Denmark).

Images were obtained using an Olympus BX61 upright fluorescent microscope (ColorView IIIU camera) and a Zeiss LSM510 through 40 X/1.30 oil DIC EC Plan-Neofluar objective lens. An argon laser of 488 nm wave length was used to excite Alexa Flour 488 (green), whereas a HeNe1 543 nm laser line was used to excite Alexa Flour 596, and Laser Diode 405 nm to excite Hoechst (blue). Pinhole was kept to 1 AU and the imaging condition set to avoid empty or saturated pixels.

### RNA isolation and cDNA synthesis

Frozen human biopsies were crushed to powder in a liquid nitrogen-cooled mortar using a pestle. Muscle tissue powder and frozen adipose tissue biopsy pieces were homogenized using TissueRuptor (Qiagen) at full speed, twice for 15 sec in 1 mL QIAzol Lysis Reagent (Qiagen), and then total RNA was isolated using miRNeasy Mini Kit (Qiagen) for muscle samples or RNeasy Lipid Tissue Mini Kit (Qiagen) for adipose tissue samples. RNA integrity and concentration were determined using Agilent RNA 6000 Nano Chips on a Bioanalyzer 2100 (Agilent Technologies Inc, Waldbronn, Germany). RNA from fat (200 ng) and muscle (500 ng) was reversely transcribed into cDNA on a Gene Amp PCR 9700 thermal cycler with the high capacity cDNA reverse transcription kit (Applied Biosystems, Foster City, CA).

### High throughput mRNA sequencing

All samples were deep sequenced using the Illumina HiSeq 2000 system with multiplex at the Norwegian Sequencing Centre (www.sequencing.uio.no), University of Oslo (Norheim et al. 2014). No batch effects were observed. cDNA sequence reads alignment was performed using Tophat v2.0.8, Samtools v0.1.18, and Bowtie v2.1.0 at default settings. The reads were initially mapped to the RefSeqGene transcriptome reference. Reads that did not match the known transcriptome reference were mapped directly to the human genome (hg19). Both the transcriptome and genome references were provided by Illumina iGenomes as of 13th of April 2013. RefSeqGene is a project of NCBI’s Reference Sequence (RefSeq). It defines reference standards for well-characterized genes that represent a “standard” allele (www.ncbi.nlm.nih.gov/refseq/rsg/about/). Postalignment quality checks were performed by converting aligned reads to Integrative Genome Viewer v2.3 tracks for visual inspection of normalized signals at any genomic location. BEDtools v2.19.1 was used to calculate coverage. Reads counted by gene feature were performed by the intersection strict mode in HTSeq v0.6.1. Read data were calculated as the number of reads mapped to each gene in the RefSeq annotation table.

### Expression analysis by quantitative real-time PCR (RT-PCR)

The cDNA reaction mixture was diluted in water and cDNA equivalent of 10 ng RNA from muscle was analyzed. Quantitative RT-PCR was performed with reagents from Applied Biosystems using 7900HT Fast instrument and SDS 2.3 software (Applied Biosystems) as previously described (Haugen et al. 2010). Predeveloped primers and probe sets (TaqMan assays, Applied Biosystems, Foster City CA) were used to analyze mRNA levels of *PLIN1* (Hs01106925_m1), *PLIN2* (Hs00605340_m1), *PLIN3* (Hs00998416_m1), *PLIN4* (Human; Hs00287411_m1 or mouse; Mm01272159_m1), *PLIN5* (Hs00965990_m1), beta-2 microglobulin (*B2M*, Hs00984230_m1) and TATA box-binding protein (*Tbp*, Mm00446971_m1). Relative target mRNA expression levels were calculated as 2^-ΔCt^, normalizing data to endogenous *B2M* or *Tbp*.

### Immunoblotting

Samples used for detection of PLIN4 protein in skeletal muscle lysates were homogenized and fractionated as previously described (Norheim et al. 2011). Briefly, muscle biopsies were taken from the middle part of *m. vastus lateralis* by three different insertions under local anesthesia from a subgroup of untrained male subjects (*n* = 3). Muscle samples were rinsed in saline and frozen in isopentane on dry ice and stored at −80°C until analysis. Muscle samples (50 mg) were homogenized and fractionated into cytosolic, membrane, nuclear, and cytoskeleton fractions using ProteoExtract subcellular proteome extraction kit (Merck KgaG, Darmstadt, Germany) according to the manufacturer’s protocol. The protein concentration of each sample was determined by BC Assay (Protein assay kit, Uptima, Montluçon, France).

Proteins from different skeletal muscle fractions (cytosol, membrane, nuclear, and cytoskeletal) were separated by SDS-PAGE (7.5%; Criterion XT Bis-tris precast gel, Bio-Rad, Hercules, CA). Then, proteins were blotted to a polyvinylidene fluoride membrane (Immobilon-P, Millipore) and blocked over night at 4°C in blocking buffer TBS-T (20 mmol/L Tris pH 7.5, 137 mmol/L NaCl, 0.1% Tween-20 (v/v) containing 3% BSA (w/v) and 0.02% sodium azide (w/v). The membrane was incubated with anti-PLIN4 (#GP34; Progen) over night at 4°C and followed by incubation with HRP-conjugated secondary antibody (Jackson ImmunoResearch Laboratories, Inc., PA). Proteins were visualized by Gel logic 2200 PRO imaging system, using an enhanced chemo-luminescence kit (SuperSignal West Dura Extended Duration Substrate, Thermo Scientific, Rockford, IL).

To make sure that all samples contained equal amounts of protein, two SDS-PAGE gels were loaded with the same amount of sample lysate. Then, one of the gels was silver stained (Pierce™ Silver Stain Kit, Thermo Scientific, Rockford, IL) whereas the other underwent western blotting as described above. Silver staining substituted the conventional way of staining the membranes by Ponceau S, because in our experiment protein on membrane did not bind to Ponceau S dye for some reason.

Antibodies binding to different cell fraction markers were used to examine the purity of the muscle cell fractions according to the ProteoExtract kit recommendation; anti-GAPDH (#ab9484, Abcam, Cambridge, UK) and anti-HSP90 (#ab59457, Abcam) were used as cytosol markers; anti-Desmin (#ab 6322, Abcam) for cytoskeleton; anti-PARP1(Ab-2) (#Am30, CalBiochem, Billerica, MA) and anti-HMGB1 (#ab77302, Abcam) as nuclear markers; and anti-Cox2 (#ab90345, Abcam) and anti-Calnexin (#208883, CalBiochem, Billerica, MA) as membrane markers. Cytosol and cytoskeleton showed no contamination from other cell fractions. The membrane fraction showed acceptable purity, whereas nuclear fraction had some contamination. However, the fact that we observed some contamination of the nuclear fraction does not affect our result as no PLIN4 was observed in the nuclear fraction.

### Statistics

EdgeR v3.4.2 was used for differential gene expression analyses to calculate normalized gene expression levels in counts per million mapped reads (CPM) and fragments per Kilobase per million fragments mapped (FPKM). Statistical significance was calculated by a mixed GLM approach. A *P*-value of <0.05 was considered statistically significant. Filtering strategies and quality checks were performed in R v3.0.3 following the edgeR instructions (Robinson et al. 2010). Bivariate correlation analyses were performed in SPSS v20.0. Pearson’s correlation coefficient was used on normally distributed parameters. Spearman’s rank correlation was used on data without normal distribution and on analyses including few samples. Data are presented as means ± SEM. Correlation analyses of all the genes detected in the mRNA-sequencing analysis were performed in R v3.0.3. The gene set of genes that correlated (*r* > 0.5) was investigated further with gene enrichment analysis. Investigation of overlaps in KEGG gene sets was done on a platform provided by the Broad Institute (http://www.broadinstitute.org/gsea/msigdb/annotate.jsp)

## Results

### Expression of perilipins before and after 12 weeks of training

**Figure 1:**
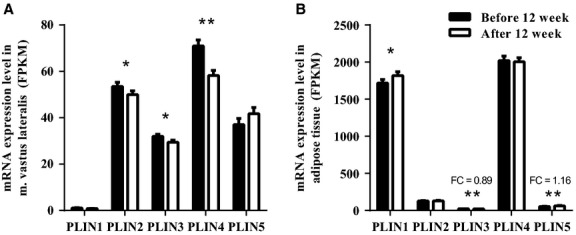
Gene expression of Perilipins (PLINs) in biopsies from *m. vastus lateralis* and adipose tissue before and after 12 weeks of training. mRNA expression was determined with high throughput sequencing and is shown as fragments per kilobase of transcript per million mapped reads (FPKM). (A) Skeletal muscle and (B) adipose tissue mRNA expression of the PLINs. Bars represent means ± SEM (*n* = 26 in muscle and *n* = 24 in adipose tissue). *P < 0.05, **P < 0.01, compared to values before training. Fold change (FC) = gene expression after 12 weeks of training/gene expression before the intervention.

To determine if 12 weeks of combined strength and endurance training intervention affected *PLIN* expression, we determined *PLIN* mRNA expression levels by high throughput mRNA sequencing in skeletal muscle (Fig.1A) and subcutaneous adipose tissue (Fig.1B), and validated the results in muscle using quantitative RT-PCR (Figure S2). Before 12 weeks training *PLIN4* had the highest mRNA expression level (71.5 FPKM) in muscle, then *PLIN2* (52 FPKM), *PLIN5* (40 FPKM), *PLIN3* (30.3 FPKM) and *PLIN1* (1.3 FPKM). The expression level of *PLIN2* (-8%), *PLIN3* (-8%), and *PLIN4* was significantly reduced (−23%, *P* < 0.01) following long-term exercise, whereas the expression of *PLIN5* tended to increase (+16%, *P* = 0.18) (Fig.1 A). Expression of *PLIN1* in skeletal muscle was very low (Fig.1A), and the weak signal may originate from intramuscular adipocytes.

In subcutaneous adipose, PLIN1 and PLIN4 were highly expressed compared to the other PLINs (Fig.1B). Before 12 weeks training PLIN4 had the highest mRNA expression level (2020 FPKM), then *PLIN1* (1717 FPKM), *PLIN2* (128 FPKM), *PLIN5* (54 FPKM), and *PLIN3* (24 FPKM). Whereas the expression level of PLIN1 (+6%, *P* < 0.05) and PLIN5 (+16%, *P* < 0.01) increased following 12 weeks of exercise, the expression of PLIN3 decreased (−11%, *P* < 0.01).

### PLIN4 is located in the sarcolemmal region

**Figure 2:**
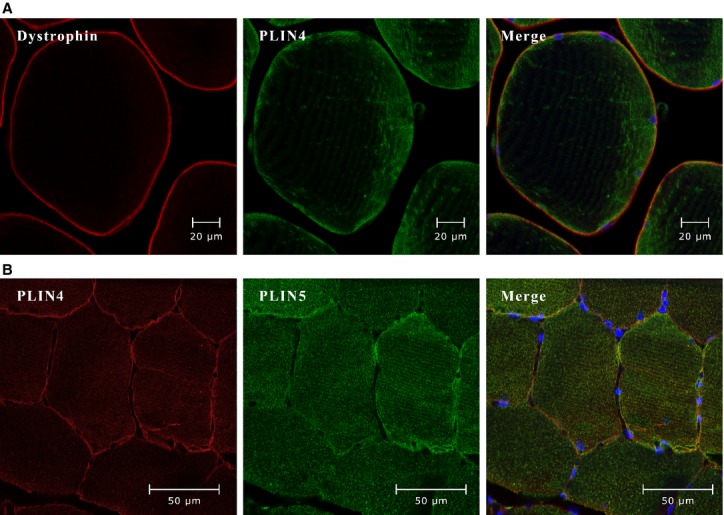
Perilipin (PLIN)4 is close to the sarcolemma of skeletal muscle fibers whereas PLIN5 is spread uniformly. (A) Representative images (630X) from confocal fluorescence microscopy of semithin (5 *μ*m) cross sections of paraffin-embedded human *m. vastus lateralis* biopsy (*n* = 10). Dystrophin (red, rabbit anti-dystrophin) binds to the outside of the sarcolemma, whereas PLIN4 (green, guinea pig anti-PLIN4) is located at or just beneath the sarcolemma. (B) The image (400X) was obtained by confocal fluorescence microscopy of sections of paraffin-embedded biopsies from *m. vastus lateralis* (*n* = 10), stained with antibodies against PLIN4 (red, rabbit anti-PLIN4) and PLIN5 (green, guinea pig anti-PLIN5).

**Figure 3:**
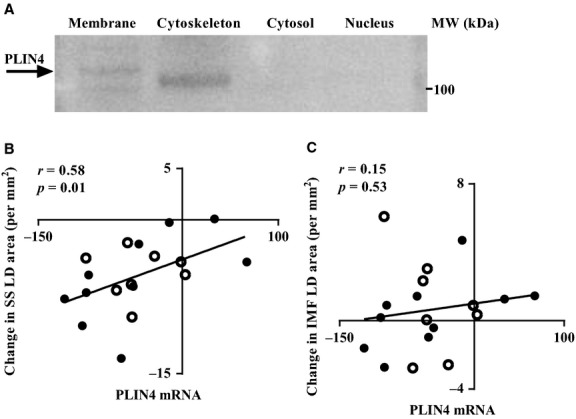
Perilipin (PLIN)4 is associated with the membrane. (A) PLIN4 protein was analyzed by western blotting in different fractions (membrane/organelles, cytoskeleton, cytosol, and nuclear) from human muscle biopsies (*n* = 3). Each lane was loaded with 8 *μ*g protein and in the membrane/organelles fraction a band was observed ∼ 120 kDa using a PLIN4-specific antibody (arrow). (B) It shows correlations between change in *PLIN4* mRNA expression and change in liquid droplets (LDs) in different subcellular locations after 12 weeks of training. mRNA expression was determined with high throughput sequencing expressed as fragments per Kilobase per million fragments mapped (FPKM). The area of LDs was calculated from LD diameters using EM images. Bivariate correlation analysis was performed between change in *PLIN4* mRNA expression levels and change in area of LDs in the SS (B) and IMF regions (C) after 12 weeks of training (*n* = 18).

We determined the cellular localization in skeletal muscle fibers of the two PLINs (PLIN4 and 5) that showed the largest changes following training. Immunospecific antibodies against dystrophin, a protein residing in the cytoplasmic layer of the sarcolemma (Watkins et al. 1988), were used as a plasma membrane marker (Fig.2A). Co-staining with antibodies detecting PLIN4 and dystrophin indicates that PLIN4 was located at or close to the sarcolemma (Fig.2A). Staining with a different antibody detecting PLIN4 also gave staining at or close to the plasma membrane (Fig.2B). Co-staining with PLIN5 showed that PLIN5 had a uniform distribution within the muscle fiber (Fig.2B). Next, we performed western blotting using protein fractions from human *m. vastus lateralis*. Antibodies against PLIN4 bound to two protein bands at ∼100–120 kDa in the membrane/organelle fraction (lane 1), whereas the cytosolic (lane 3) and nuclear (lane 4) protein fractions showed no positive signal for PLIN4 (Fig.3A). In the cytosolic protein fraction a band of ∼100 kDa was observed. The immunoreactive band at ∼120 kDa in the membrane/organelle fraction corresponds best to the calculated molecular weight of PLIN4 (134 kDa). The samples were analyzed with SDS-PAGE and silver staining to control that all fractions had the same amount of protein (Figure S3).

### Correlation between *PLIN4* expression and SS LD populations

To determine if the reduction in PLIN4 by long-term training correlated with the previously reported reduction of LDs in the SS-region of skeletal muscle (Li et al. 2014), we performed bivariate correlation analyses between the training-induced changes in *PLIN4* mRNA and the corresponding changes in the two populations of LDs (SS and IMF). The *PLIN4* mRNA level was measured on the same set of samples as the LDs reported in our previous study (Li et al. 2014). We observed a strong and significant correlation of *PLIN4* mRNA with the SS LDs (*r* = 0.58, *P* = 0.01, Fig.3B) but not with the IMF LDs (*r* = 0.15, *P* = 0.53, Fig.3C). Considering the absolute values, we found a trend toward a positive correlation between *PLIN4* mRNA and the area of SS LDs before as well as after 12 weeks of training (*r* = 0.44, *P* = 0.07, and *r* = 0.41, *P* = 0.09, respectively; Figure S4A and C). *PLIN4* expression was positively correlated with the area of IMF LDs (*r* = 0.47, *P* < 0.05, Figure S4B) before training, but we found only a trend after 12 weeks of training (*r* = 0.40, *P* = 0.09, Figure S4D).

For the other PLINs we observed a significant correlation between changes of mRNA expression of *PLIN3* and *PLIN5* with changes in area of IMF LDs (*r* = 0.63, *P* < 0.01, and *r* = 0.48, *P* < 0.05, respectively; data not shown). No correlations were observed between *PLIN2* expression and LDs areas in SS and IMF.

### *PLIN4* mRNA is associated with genes encoding proteins related to fatty acid oxidation and phospholipid biosynthesis

**Figure 4:**
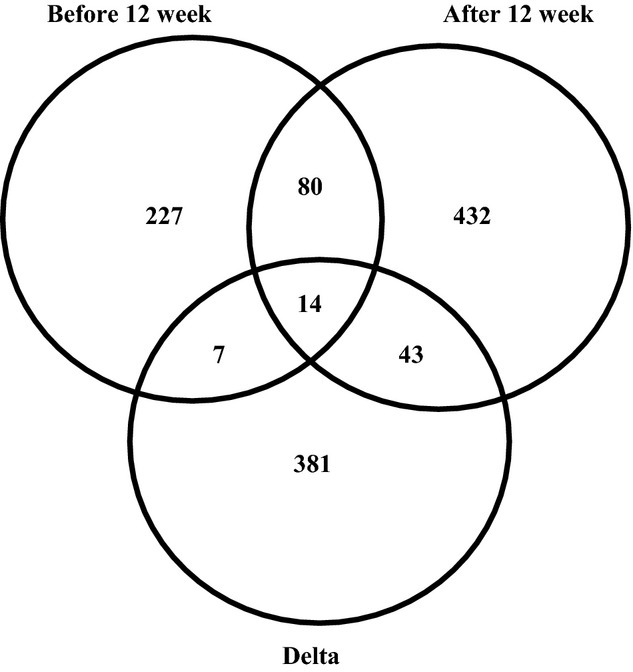
Correlation analyses between the expression of *PLIN4* and all other genes detected by mRNA sequencing in biopsies from *m. vastus lateralis*. The expression levels were correlated using Spearman’s test at before and after 12 weeks of training, and the change following long-term exercise (before 12 weeks – after 12 weeks). The Venn diagram shows the number of genes with correlation coefficients *r* > 0.5, and the overlapping areas represent the number of genes that are correlated in two or all data sets.

Because both the cellular localization of *PLIN4* (Fig.2) and its reduced expression in response to long-term exercise (Fig.1) correlate with the reduced number of LDs in the SS-region, we screened for genes exhibiting the highest correlations (*r* > 0.5) with *PLIN4* expression in samples collected before (328 genes) and after 12 weeks of training (569 genes), and the change in expression following long-term exercise (445 genes) (Fig.4). Of these genes 14 consistently exhibited correlation with *PLIN4* mRNA in all three data sets (Table1). Two of these genes are known to code for proteins that increase fatty acid oxidation, namely *CPT1B* (carnitine palmitoyl transferase 1B) and *MLYCD* (malonyl-CoA decarboxylase), and two genes are linked to increased *de novo* phospholipid biosynthesis (*AGPAT2* and *AGPAT3*; 1-acylglycerol-3-phosphate O-acyltransferase). A pathway analysis of gene-expressions showing a correlation (*r* > 0.5) in two or more data sets, was performed to compute overlaps in KEGG gene sets (Table S1). Our analysis confirmed that *PLIN4* mRNA expression is associated with fatty acid metabolism, and the two most prominent pathways were propionate and fatty acid metabolism.

**Table 1:** Gene expression correlated to PLIN4 mRNA expression in three data sets. The expression of PLIN4 was correlated to the expression of all other genes (*n = *21000) detected by mRNA sequencing in biopsies from *m. vastus lateralis* (*n* = 26). The correlation coefficients were obtained before, after 12 weeks of training and the change following long-term exercise (after 12 week – before)

Gene symbol	Gene name	FPKM*	Before 12 week		After 12 week		Delta	
			*r* †	*P* ‡	*r*	*P*	*r*	*P*
FAM50B	Family with sequence similarity 50, member B	12.3	0.74	2E-05	0.75	1E-05	0.60	1E-03
CPT1B	Carnitine palmitoyltransferase 1B	0.6	0.69	1E-04	0.76	7E-06	0.73	3E-05
AGPAT2	1-acylglycerol-3-phosphate O-acyltransferase 2	10.1	0.64	5E-04	0.64	4E-04	0.50	9E-03
EMD	Emerin	35.8	0.59	2E-03	0.61	1E-03	0.57	3E-03
WBP2	WW domain binding protein 2	38.1	0.58	2E-03	0.66	3E-04	0.51	7E-03
SPATA2	Spermatogenesis associated 2	3.5	0.57	3E-03	0.57	2E-03	0.53	6E-03
GRB10	Growth factor receptor-bound protein 10	14.4	0.56	3E-03	0.64	4E-04	0.63	5E-04
PLIN5	Perilipin 5	40.9	0.56	3E-03	0.62	8E-04	0.71	4E-05
TEAD3	TEA domain family member 3	12.2	0.56	3E-03	0.74	2E-05	0.52	7E-03
UBAP2	Ubiquitin associated protein 2	12.7	0.55	4E-03	0.57	3E-03	0.62	7E-04
WSCD1	WSC domain containing 1	3.2	0.54	4E-03	0.64	4E-04	0.63	6E-04
AGPAT3	1-acylglycerol-3-phosphate O-acyltransferase 3	17.4	0.53	5E-03	0.68	1E-04	0.51	8E-03
MLYCD	Malonyl-CoA decarboxylase	24	0.53	5E-03	0.51	7E-03	0.64	5E-04
KREMEN1	Kringle containing transmembrane protein 1	7.5	0.52	7E-03	0.57	2E-03	0.59	2E-03

* Fragments per kilobase of transcript per million mapped reads. Mean of 6 time points.

† The correlations were obtained by using Spearman’s test (*r* > 0.5)

‡ *P*-value obtained by using Spearman’s test

### Correlation between *PLIN4* mRNA expression and lipid species

We applied high-performance liquid chromatography with ELSD to quantify free fatty acids (De Ferranti and Mozaffarian 2008), TAG, CE, cholesterol, and different phospholipids; lysophosphatidylethanolamine (LPE), phosphatidylethanolamine (PE), phosphatidylinositol (PI), phosphatidylcholine (PC) and sphingomyelin (SPM) in skeletal muscle biopsies from *m. vastus lateralis* before and after 12 weeks of training intervention. *PLIN4* mRNA levels showed a positive correlation with levels of PE (*r* = 0.41, *P* = 0.04) and PC (*r* = 0.48, *P* = 0.01) after 12 weeks of training (Table2). The changes in levels of PE (*r* = 0.38, *P* = 0.06) and PC (*r* = 0.37, *P* = 0.07) values during intervention showed a borderline significant correlation with changes in *PLIN4* mRNA (Table2).

**Table 2:** Correlation between *PLIN4* mRNA levels and concentration of intramuscular lipid species. The mRNA expression level of *PLIN4* was correlated with the content of different lipid species in biopsies from *m. vastus lateralis* (*n* = 26) before, after 12 weeks of training and the change following long-term exercise (after 12 week - before)

	Before 12 week		After 12 week		Delta	
	*r* *	*P* †	*r*	*P*	*r*	*P*
Cholesterol Esters	0.07	0.75	0.09	0.68	0.03	0.88
Triacylglycerol	0.21	0.31	0.20	0.33	0.19	0.36
Free fatty acids	0.19	0.37	0.23	0.25	0.25	0.23
Cholesterol	−0.05	0.83	0.26	0.20	0.15	0.48
Total neutral	0.21	0.31	0.20	0.34	0.15	0.49
Phosphatidylethanolamine	0.23	0.26	0.41	0.04	0.38	0.06
Lysophosphatidylethanolamine	0.02	0.91	0.01	0.95	−0.30	0.14
Phosphatidylinositol	0.09	0.72	0.13	0.55	−0.05	0.85
Phosphatidylcholine	0.27	0.19	0.48	0.01	0.37	0.07
Sphingomyelin	0.12	0.56	0.18	0.37	0.08	0.70
Lysophosphatidylcholine	0.13	0.52	0.09	0.66	−0.02	0.94
Total polar	0.21	0.31	0.32	0.11	0.36	0.08
Total lipids	0.32	0.11	0.36	0.07	0.23	0.27

* The correlations were obtained by using Spearman’s test (*r* > 0.5)

† *P*-value obtained by using Spearman’s test

### PLIN4 expression in slow- and fast-twitch muscle fibers

**Figure 5:**
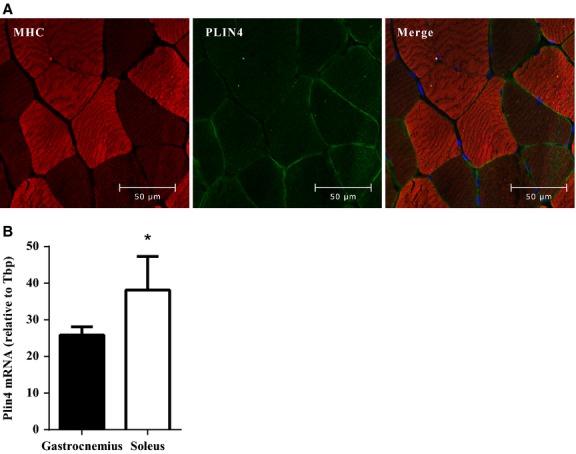
Perilipins (PLIN)4 is mostly expressed in slow muscle fibers. (A) Representative images (400X) from confocal fluorescence microscopy of sections of paraffin-embedded biopsies from *m. vastus lateralis* (*n* = 10), stained with antibodies against myosin heavy chain fast (red, mouse anti-myosin heavy chain; skeletal, fast) and PLIN4 (green, guinea pig anti-PLIN4). Fast muscle fibers are stained in bright red color, whereas slow fibers appear as dark. (B) *M. soleus* and *m. gastrocnemius* were dissected from male C57BL/6N mice (*n* = 5) and *Plin4* mRNA expression was determined by quantitative RT-PCR. Plin4 mRNA was normalized to *Tbp* mRNA. Results are presented as means ± SEM, *P < 0.05 (two-tailed Student’s *t*-test).

To evaluate further the localization of PLIN4 in skeletal muscle, we used antibodies directed against the fast-twitch myosin heavy chains and PLIN4 to obtain images by immunofluorescence microscopy. We observed thicker PLIN4 rims at the periphery of the unstained muscle fibers by the antibody directed against the fast-twitch types of myosin heavy chain (Fig.5A), indicating that more PLIN4 exists in slow-twitch fibers. This finding was further supported by gene expression data from murine *m. soleus* and *m*. *gastrocnemius,* showing significantly higher *Plin4* mRNA expression in *m. soleus* (Fig.5 B) with most oxidative slow-twitch fibers. Our results provide evidence that PLIN4 is more highly expressed in oxidative slow-twitch muscle fibers than in fast-twitch fibers.

### Intramuscular adipocytes

**Figure 6:**
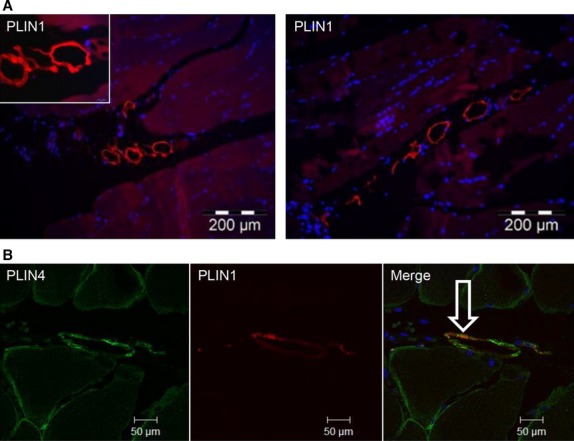
Intramuscular adipocytes. (A) Sections of paraffin-embedded biopsies from *m. vastus lateralis* (*n = *8) were stained with PLIN1 antibody (red, mouse anti-PLIN1) to detect adipocytes (A). Representative images (200X) were obtained by wide-field fluorescence microscopy. High magnification (1000X) of adipocytes is shown in the insert. (B) The sections were stained for PLIN4 (green, rabbit anti-PLIN4) and PLIN1 (red, mouse anti-PLIN1), showing partly co-localization in intramuscular adipocytes (*n* = 4). The merged image (200X) shows co-localization of the proteins (white arrow). The image was obtained by confocal fluorescence microscopy.

We observed intramuscular adipocytes by using an antibody targeting PLIN1 (Fig.6 A), which binds to LDs in adipocytes (Greenberg et al. 1991). By using Toluidine blue staining on osmium-fixed paraffin-embedded muscle biopsies we also observed intramuscular adipocytes (Figure S5). TAGs are extracted during fixation and LDs appear as hollow structures in immunohistochemistry images. Using antibodies targeting PLIN1 and PLIN4 we observed co-staining of PLIN4 and PLIN1 in adipocytes in the intramuscular interstitium (Fig.6 B).

## Discussion

We observed that PLIN4 is located close to the plasma membrane/SS-region, in contrast to PLIN5 that show an even distribution across the whole fiber. The reduction of *PLIN4* mRNA in skeletal muscle in response to long-term training is associated with the decrease in LDs size in the SS-region, which links reduction in *PLIN4* mRNA to a reduction in LDs in the SS-region.

Several lines of evidence suggest that the majority of PLIN4 is physically linked to the sarcolemma or found in the SS region. Firstly, we demonstrate for the first time that PLIN4 is located close to the sarcolemma using immunofluorescence microscopy (Fig.2A and B). Secondly, by western blotting we identified PLIN4 in the membrane/organelle fraction of skeletal muscle homogenates. Finally, we observed a positive correlation between changes in *PLIN4* mRNA expression level and change in size of SS LDs in response to long-term training (Peters et al. 2012). McPherson et al. studied LDs and PLIN5 protein content in resting and electro-stimulated rat *m. soleus* and observed more LDs in resting muscles as compared to the stimulated muscle (MacPherson et al. 2011). However, no difference was found between the resting and electrostimulated muscles concerning PLIN5 protein content suggesting that PLIN5 is not recruited to LDs in response to muscle fiber contraction (MacPherson et al. 2011). Peters and colleagues reported a correlation between the size of LDs in muscle and PLIN5 at protein level but not for PLIN4. In contrast to their study, we analyzed the two distinct LD populations separately, which might explain the different results. As outlined in a previous study (Li et al. 2014), the LD populations in IMF and SS respond differently to long-term training. Thus, we hypothesize that PLIN4 is mainly located to SS LDs and these LDs are more metabolically responsive to physical activity than IMF LDs.

We show that *PLIN4* is the most highly expressed PLIN in skeletal muscle, and the most regulated PLIN in response to long-term combined endurance- and strength-training. Peters et al. found no effect of 12 weeks of endurance training on *PLIN4* mRNA level and protein content in skeletal muscle (Peters et al. 2012). The apparent discrepancy between these observations may be due to differences in exercise protocols. Thus, Gjelstad et al. have shown that 10 weeks of strength training intervention tended to reduce *PLIN4* expression in exercised muscle (Gjelstad et al. 2012). The discrepancies might also be explained by differences in effects of the exercise interventions on SS LDs, phospholipid composition or amount of slow muscle fibers, which again might affect expression of PLIN4. We monitored *mRNA* expression; it is possible that a reduction in *PLIN4* mRNA may not promote lower PLIN4 protein levels. Unfortunately, limited access to muscle protein lysates made us unable to measure protein concentrations of the different PLINs in muscle biopsies before and after the training intervention.

The tendency of an increased level of *PLIN5* mRNA in muscle after long-term training (Fig.1A) is in accordance with the observation of Shepherd et al. demonstrating that sprint interval and traditional endurance training increased net intramuscular triacylglycerol breakdown and expression of PLIN2 and 5 (Shepherd et al. 2013). Two other long-term endurance training studies reported increase in PLIN5 protein content despite no change in mRNA levels (Peters et al. 2012; Louche et al. 2013). The recent study of Ramos et al. suggests that the increased level of PLIN5 protein often observed after long-term exercise is not caused by PLIN5 in the mitochondrial fraction of skeletal muscle (Ramos et al. 2015). PLIN5 is reported to be enhanced when intramyocellular triacylglycerol levels or hydrolysis are high (Shepherd et al. 2013). We observed increased level of *PLIN5* mRNA in skeletal muscle after 12 weeks of training, although the subjects had significantly reduced SS LDs (Li et al. 2014). This is in accordance with the study of Louche et al. (Louche et al. 2013) reporting increased PLIN5 protein content despite reduced intramyocellular triacylglycerol levels after 8 weeks of endurance training. Our results suggest that transcriptional regulation of *PLIN5* mRNA is enhanced by exercise independent of the amount of LDs accumulated in the SS-region.

The size of LDs may change rapidly in response to cellular energy metabolism. Because the sizes of LDs increase within hours during lipid storage, substantial amounts of phospholipids are required to coat the LDs. Conversely, LDs may shrink during fatty acid oxidation. *In vitro* experiments on S2 cells have shown that oleate loading activates PC synthesis by activation of the phosphocholine cytidylyltransferase (CTP) on the surface of LDs (Krahmer et al. 2011). Removing oleate from the medium of S2 cells reduced the activity of CTP (Krahmer et al. 2011). The effect of increased content of PC in the cell membrane of LDs might be to prevent LD coalescence, which otherwise yields accumulation of large lipolysis-resistant LDs (Krahmer et al. 2011). We observed positive correlations between *PLIN4* mRNA and volume of SS LDs, genes involved in de novo phospholipid biosynthesis, and the concentration of the phospholipids PE and PC. Future studies should focus on whether PLIN4 may affect phospholipid synthesis or vice versa. It is also possible that the correlation we find between PLIN4 and phospholipid biosynthesis depends on other factors such as SS LDs volume.

Acute as well as long-term exercise may have an effect on adipose tissue. During an acute exercise bout adipose tissue is the major source of circulating FAs required to supply the skeletal muscles with energy (Horowitz 2003). Long-term training can reduce adipose tissue size and activate signaling pathways in visceral adipose tissue such as AMPK (Takekoshi et al. 2006). We observed a small but significant effect of long-term training on several PLINs. Whereas the mRNA expression of *PLIN1* and *PLIN5* increased in response to the intervention, the expression of *PLIN3* decreased. The effect of training on several PLINs could be due to a reduction in total body fat of ∼7% as measured with MRI (Langleite TM, submitted, 2015). It is also noteworthy that PLIN4 is the PLIN with the highest mRNA expression level in both skeletal muscle and subcutaneous adipose tissue.

Skeletal muscles contain two major fiber types, type I oxidative fibers (slow-twitch) and type II glycolytic fibers (fast-twitch). Oxidative type I fibers store more lipids than glycolytic type II fibers (Dyck et al. 1997). The level of PLIN2 is approximately two-fold higher in type I than type II fibers (Shaw et al. 2009; Shepherd et al. 2012). Wolins et al. demonstrated that PLIN5 is highly expressed in heart and *m. soleus,* which both mostly are composed of type I fibers (Wolins et al. 2006; Dalen et al. 2007). Our immunohistochemistry analyses indicated that PLIN4 is more abundant in type I than type II fibers. In accordance with these results, a higher level of *Plin4* mRNA is expressed in murine *m. soleus* as compared to *m*. *gastrocnemius* (Fig.5 B). *M. soleus* contains mostly type I fibers, whereas *m*. *gastrocnemius* has a mixture of type I and II fibers (Soukup et al. 2002). Moreover, soleus muscles tend to accumulate more subsarcolemmal LDs (MacPherson et al. 2011) as compared to muscles with predominantly type II fibers.

The low level of *PLIN1* mRNA expression in the muscle biopsies might be explained by PLIN1 being relatively specific to adipocytes (Greenberg et al. 1991). In support of this, we show that PLIN1 is highly expressed in subcutaneous adipose tissue. Intramuscular adipocytes represent a depot of lipids between muscle bundles (Vettor et al. 2009), and they are present along the blood vessels (Yudkin et al. 2005; Britton and Fox 2011). Our immunofluorescence images show colocalization of PLIN1 and PLIN4s in intramuscular structures, which we interpret as adipocytes (Fig.6B). We also observed intramuscular adipocytes when using Toluidine blue staining on osmium-fixed paraffin-embedded muscle biopsies (Figure S5).

In summary, we observed that *PLIN4* mRNA in muscle is modestly reduced after 12 weeks of training and its reduction in expression correlates with reduced expression of genes involved in de novo phospholipid biosynthesis and the cellular content of PE and PC. We have also shown that PLIN4 localizes to the periphery of muscle fibers, and we saw a correlation between the changes in *PLIN4* expression with changes in the area of SS LDs in response to the training intervention. Moreover, we observed that PLIN4 is localized to intramuscular adipocytes, and is more highly expressed in slow-twitch muscle fibers as compared to fast-twitch. Overall, these studies indicate that PLIN4 is an exercise-responsive PLIN, which is mainly localized on SS LDs in muscle fibers with effects on de novo phospholipid biosynthesis.

## Supporting Information

**Figure S1.** Electron micrograph of lipid droplets (LDs) in two distinct regions of skeletal muscle fibers. The subsarcolemmal (SS) LDs are located beneath the sarcolemma, whereas the intramyofibrillar (IMF) LDs are located inside the muscle fiber.

**Figure S2.** mRNA expression of *PLIN1-5* from *m. vastus lateralis* before and after 12 weeks of training. Samples from skeletal muscle biopsies were processed for mRNA expression analyses by quantitative RT-PCR using primers specific for the indicated genes. All expression data were normalized to *B2M* (beta-2-microglobulin) mRNA. Bars depict means ± SE. **P *< 0.05 and ***P *< 0.01.

**Figure S3.** Silver staining of the SDS-PAGE. The muscle cell fractions loaded on SDS gel and stained with silver. In each lane 8 *μ*g of protein loaded, 1 represents cell membrane, 2 cytoskeleton, 3 cytosol and 4 nucleus.

**Figure S4.** Correlation between *PLIN4* mRNA expression and volumes of LDs in the subsarcolemmal (SS) and intramyofibrillar (IMF) regions in skeletal muscle. mRNA expression was determined with high throughput sequencing as FPKM. The area of LDs was calculated from LD diameters using electron microscopy images. Bivariate correlation analysis was performed between *PLIN4* mRNA expression and area of LDs in SS (A, C) and IMF regions (B, D) before and after 12 weeks of training.

**Figure S5.** Osmium fixed paraffin-embedded section of biopsies from *m. vastus lateralis* were stained using Toluidine blue to detect intramuscular adipocytes (*n* = 8).

**Table S1.** Pathway analysis of genes correlating with *PLIN4*. Genes with medium to high correlation (*r *> 0.5) with *PLIN4* mRNA in two or more time points were selected and analyzed for overlaps in KEGG pathway gene sets (*n* = 26).
