# Supplementary figures and images for: Perilipin 4 in human skeletal muscle: localization and effect of physical activity
Source: Physiol Rep. 2015 Aug 11;3(8):e12481. doi: 10.14814/phy2.12481 (PMC4562567; doi:10.14814/phy2.12481)

**Supplementary Figure 1**

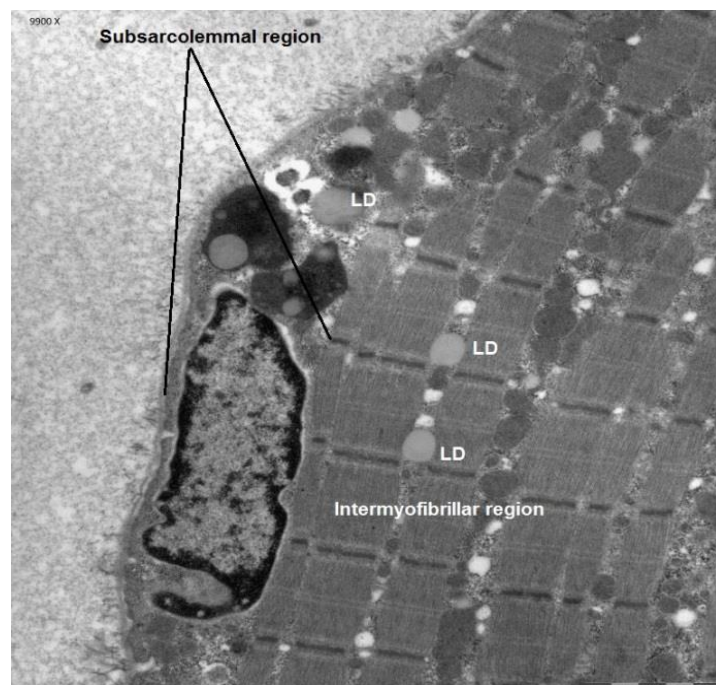

Supplement: Supplementary file 1 [file phy20003-e12481-sd1.pdf]

**Supplementary Figure 2**

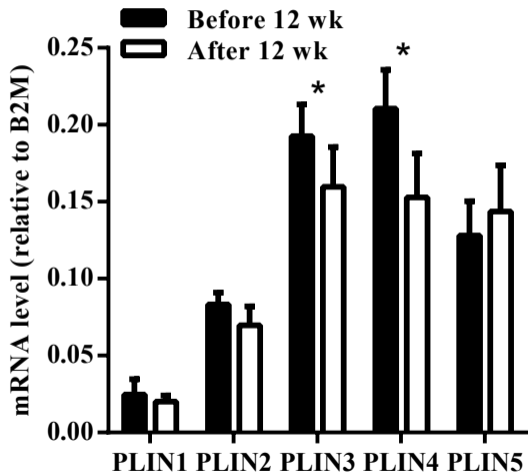

Supplement: Supplementary file 2 [file phy20003-e12481-sd2.pdf]

**Supplementary Figure 3**

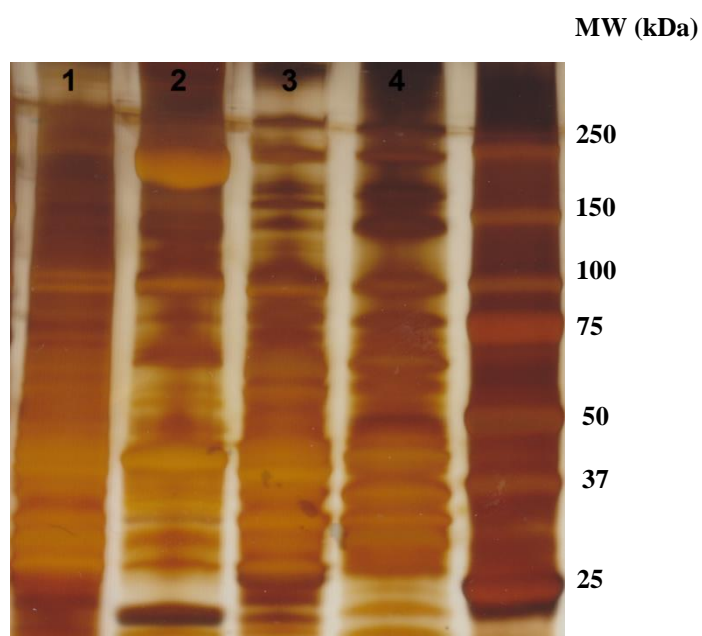

Supplement: Supplementary file 3 [file phy20003-e12481-sd3.pdf]

Supplementary Figure 4

Before 12 wk

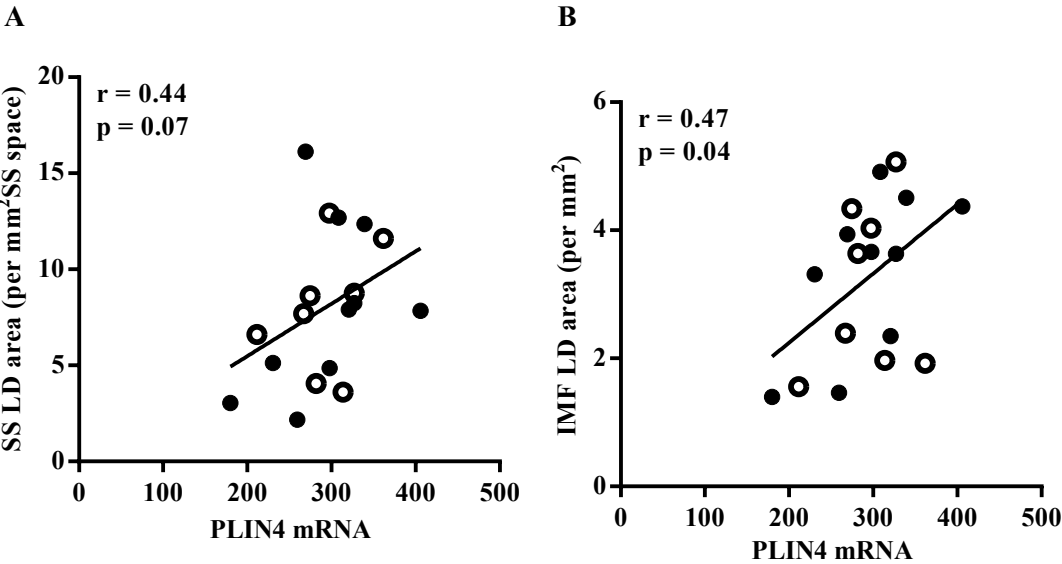

After 12 wk

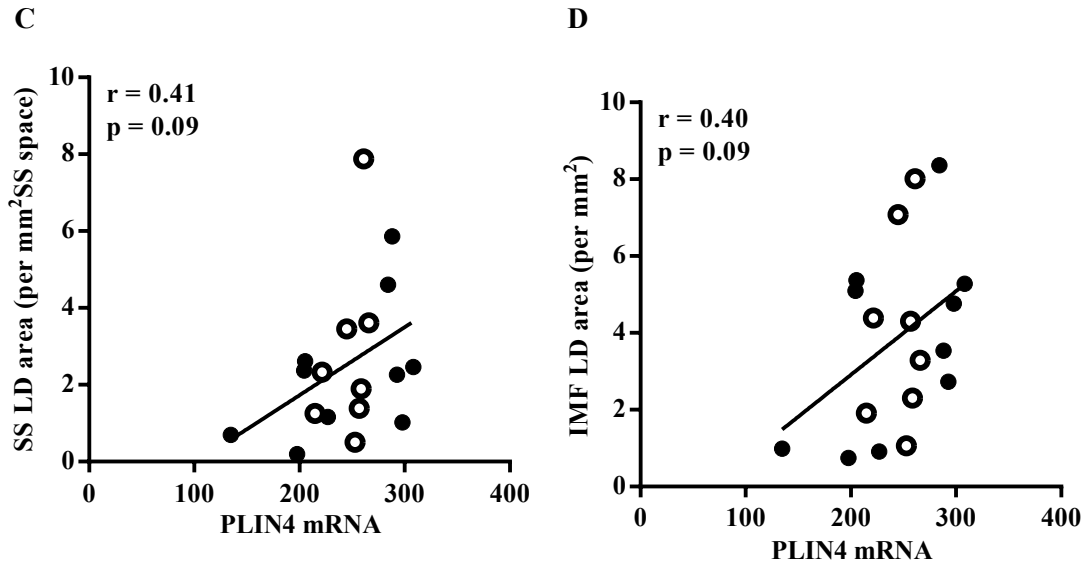

Supplement: Supplementary file 4 [file phy20003-e12481-sd4.pdf]

**Supplementary Figure 5**

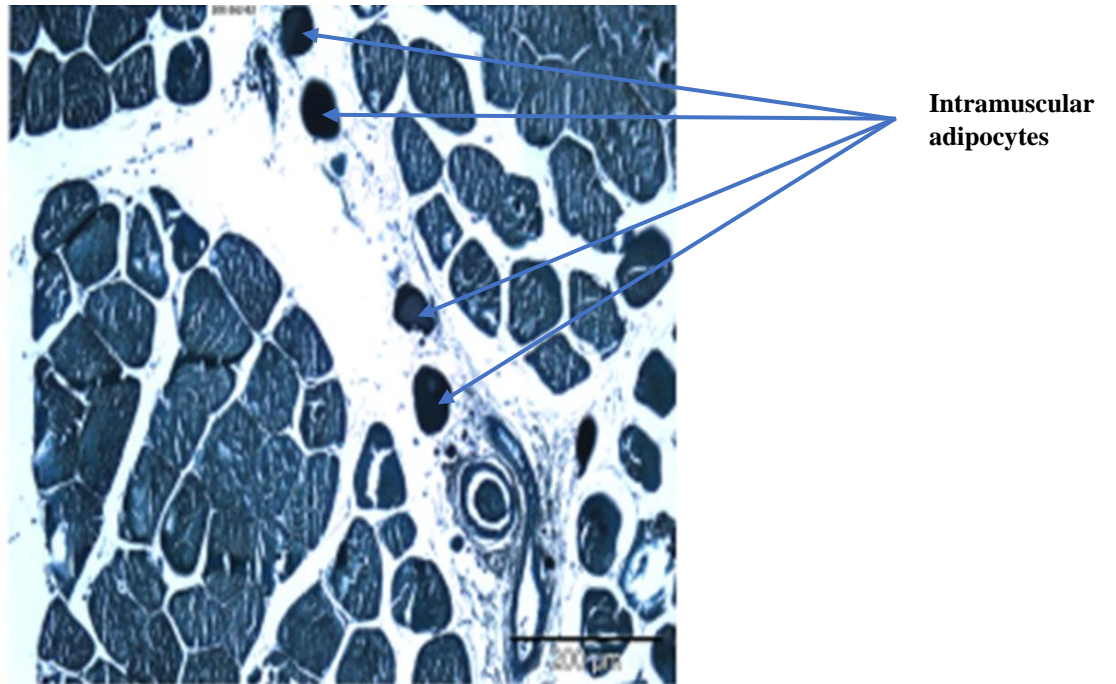

Supplement: Supplementary file 5 [file phy20003-e12481-sd5.pdf]
