# Supplementary material for: Perilipin 4 in human skeletal muscle: localization and effect of physical activity
Source: Physiol Rep. 2015 Aug 11;3(8):e12481. doi: 10.14814/phy2.12481 (PMC4562567; doi:10.14814/phy2.12481)
Supplement: Supplementary file 6 [file phy20003-e12481-sd6.docx]

**Supplementary Table 1. Pathway analysis of genes correlating with *PLIN4*.** Genes with medium to high correlation (r>0.5) with *PLIN4* mRNA in two or more time points were selected and analyzed for overlaps in KEGG pathway gene sets (n=26).

| **KEGG gene set** | **Overlapping genes** | **#Genes in gene set (K)** | **#Genes in overlap (k)** | **k/K** | **P^1^** | **q^2^** |
| --- | --- | --- | --- | --- | --- | --- |
|  |  |  |  |  |  |  |
| Propanoate metabolism | ALDH2, HADHA, MLYCD, ACACB, ACSS2 | 33 | 5 | 0.15 | 5E-08 | 8E-06 |
| Fatty acid metabolism | ALDH2, HADHA, CPT1B, ACOX1, CPT2 | 42 | 5 | 0.12 | 2E-07 | 1E-05 |
| Arginine and proline metabolism | ALDH2, MAOB, ASS1, CKM | 54 | 4 | 0.07 | 2E-05 | 1E-03 |
| beta-Alanine metabolism | ALDH2, HADHA, MLYCD | 22 | 3 | 0.14 | 4E-05 | 2E-03 |
| Insulin signaling pathway | ACACB, ARAF, PHKA2, INSR, LIPE | 137 | 5 | 0.04 | 6E-05 | 2E-03 |
| Histidine metabolism | ALDH2, MAOB, METTL2B | 29 | 3 | 0.1 | 9E-05 | 3E-03 |
| Pyruvate metabolism | ALDH2, ACACB, ACSS2 | 40 | 3 | 0.08 | 2E-04 | 5E-03 |
| Tryptophan metabolism | ALDH2, HADHA, MAOB | 40 | 3 | 0.08 | 2E-04 | 5E-03 |
| Lysine degradation | ALDH2, HADHA, DOT1L | 44 | 3 | 0.07 | 3E-04 | 6E-03 |
| Limonene and pinene degradation | ALDH2, HADHA | 10 | 2 | 0.2 | 4E-04 | 7E-03 |
| ^1^P-value computed by a hypergeometric test | | | |  |  |  |
| ^2^False discovery rate | | | |  |  |  |
